# *SLTAB2* is the paramutated *SULFUREA* locus in tomato

**DOI:** 10.1093/jxb/erw096

**Published:** 2016-03-08

**Authors:** Quentin Gouil, Ondřej Novák, David C. Baulcombe

**Affiliations:** ^1^Department of Plant Sciences, University of Cambridge, Cambridge, UK; ^2^Laboratory of Growth Regulators, Centre of the Region Haná for Biotechnological and Agricultural Research, Institute of Experimental Botany AS CR and Faculty of Science of Palacký University, CZ-78371 Olomouc, Czech Republic

**Keywords:** Auxin, DNA methylation, paramutation, photosynthesis, RdDM, siRNA, *SULFUREA*, VIGS.

## Abstract

The *sulfurea* (*sulf*) allele is a silent epigenetic variant of a tomato (*Solanum lycopersicum*) gene affecting pigment production. It is homozygous lethal but, in a heterozygote *sulf*/+, the wild-type (wt) allele undergoes silencing so that the plants exhibit chlorotic sectors. This transfer of the silenced state between alleles is termed paramutation and is best characterized in maize. To understand the mechanism of paramutation we mapped *SULF* to the orthologue *SLTAB2* of an *Arabidopsis* gene that, consistent with the pigment deﬁciency, is involved in the translation of photosystem I. Paramutation of *SLTAB2* is linked to an increase in DNA methylation and the production of small interfering RNAs at its promoter. Virus-induced gene silencing of *SLTAB2* phenocopies *sulf,* consistent with the possibility that siRNAs mediate the paramutation of *SULFUREA*. Unlike the maize systems, the paramutagenicity of *sulf* is not, however, associated with repeated sequences at the region of siRNA production or DNA methylation.

## Highlight

The chlorotic phenotype of the paramutated *sulfurea* tomato plants is due to the epigenetic silencing of the *SLTAB2* gene, involved in the translation of photosystem I.

## Introduction

Paramutation involves the transfer of epigenetic marks from a (paramutagenic) silent gene to the active (paramutable) allele so that it becomes heritably silent and paramutagenic. Several plant species exhibit paramutation and the best characterized examples, the *b1* and *pl1* loci in maize, have been linked to the process of RNA-directed DNA methylation (RdDM) (Chandler and Stam, 2004; Hollick, 2012) in which paramutagenic small interfering (si)RNAs mediate silencing of the paramutable allele. This simple model does not, however, explain why most siRNA loci are not paramutagenic: there must be other factors.

To shed light on the mechanism of paramutation we are analysing the tomato *SULFUREA* (*SULF*) locus. The silent *sulf* allele has a chlorotic phenotype (Hagemann, 1958) that is associated with reduced auxin (Ehlert *et al.*, 2008). A *sulf* homozygote is seedling lethal but a viable heterozygous *sulf*/+ plant has large chlorotic sectors that are due to paramutation of the active allele to a silenced state in early development. This system is like classic maize paramutation because the paramutated state is heritable and paramutagenic (Hagemann, 1969). *SULF* maps to the pericentromeric heterochromatin of chromosome 2, at approximately 29 cM from the *S* locus (Solyc02g077390, compound inﬂorescence) (Hagemann and Snoad, 1971) but the affected gene could not be mapped precisely due to low recombination frequency in this region (The Tomato Genome Consortium, 2012).

A gene orthologue of the *Arabidopsis ATAB2* is strongly down-regulated in chlorotic sectors of *sulf*/+ tomato (Ehlert *et al.*, 2008) but it was previously excluded as the *SULF* gene because it is still expressed at detectable levels. However, from analysis of transcriptome, methylome, and small RNA populations of wild-type and paramutated tomato leaves we show here that paramutation of *SLTAB2* is responsible for the *sulf* chlorosis and the decrease in auxin levels. *SLTAB2* silencing was associated with changes in DNA methylation and siRNA levels at its promoter, a signature of RdDM.

Additional evidence supporting the identification of *SLTAB2* as *SULF* is from virus-induced gene silencing (VIGS) of the *SLTAB2* promoter resulting in methylation of the target DNA sequence, silencing of its expression, and a phenocopy of the *sulf* chlorosis. Together, these results support a causal role of siRNAs and RdDM in paramutation but, unlike the maize examples, the *SLTAB2*/*SULF* locus lacked repeated sequences. Mapping of *SULF* to *SLTAB2* and further comparison with maize will help build a general model of paramutation in plants.

## Materials and methods

### Plant material and growth conditions

*Atab2* T-DNA knockouts (GABI-KAT line 354B01) and wild-type Col-0 seedlings were sown on 1/2 strength Murashige–Skoog medium, 1× Nitsch&Nitsch vitamins, 0.8% agar, 1.5% sucrose, pH 6; stratified for 72h at 4 °C in the dark and transferred to short-day conditions (8h light at 23 °C and 50 µmol photons m^–2^ s^−1^, 16h dark at 21 °C). Whole seedlings were collected after 7 d of growth. Tomato plants were raised from seeds in compost (Levington M3) and maintained in a growth room at 23 °C with 16/8h light/dark periods with 60% relative humidity, at a light intensity of 150 µmol photons m^–2^ s^−1^. Young leaves were collected from 1-month-old plants. *Sulf* and *sulf*/+ tissue was collected from *sulf*/+ plants that had both fully yellow (*sulf*) and fully green (*sulf*/+) sectors.

### Transcriptome analysis

Total RNA samples were prepared from 100mg of leaf tissue using TRIzol (LifeTechnologies). For qPCR, 5 µg of total RNA was first DNase treated using Turbo DNase (Ambion), following the manufacturer’s guidelines. cDNA was then synthesized using random hexamers and Oligo(dT) and SuperScript III (LifeTechnologies), according to the protocol. qPCR was performed on a Roche LC480 with SYBR in technical triplicates. mRNA abundance was normalized by the geometric mean of two housekeeping genes *TIP41* and *EXPRESSED* (Coker and Davies, 2003). Genotyping of ampliﬁed cDNA was performed by digesting 100ng puriﬁed *SLTAB2* amplicon with BaeI (NEB) for 12h at 25 °C in 1× NEB2.1, 100 µg ml^−1^ BSA, and 20 µM SAM as per the manufacturer’s instructions, and electrophoresis on a 1.5% agarose gel. Strand-speciﬁc RNA-Seq libraries for two wild-types and three pairs of *sulf* and *sulf*/+ were made and indexed with the ScriptSeq v2 kit (Epicentre) according to the protocol after RiboZero treatment (Plant leaf, Epicentre), and sequenced as a pool on one lane of HiSeq 2000 100PE. Sequences were trimmed and ﬁltered with Trim Galore! with default parameters and 11–29 million reads per library were concordantly aligned on Heinz genome SL2.50 and ITAG2.4 gene models using TopHat2 v2.0.13 (Kim *et al.*, 2013) (with parameters -r 200 --mate-std-dev 100 -N 3 --read-edit-dist 3 --library-type fr-ﬁrststrand --solexa1.3-quals, and version 2.2.4.0 of Bowtie2; alignment rate 66–73%; see Supplementary Table S1 at *JXB* online). Differential expression analysis was performed on raw counts on annotated mRNAs (ITAG2.4) with DESeq2 v1.8.1 (Love *et al.*, 2014). Genes were considered differentially expressed when the adjusted *P*-value was <0.05. Hierarchical correlation clustering of the genes differentially expressed between wt and *sulf* was performed in SeqMonk (v0.32.0). Gene Ontology analysis was performed with the goseq package (v1.20.0, Young *et al.*, 2010) using previously published gene ontology annotation (Koenig *et al.*, 2013), normalizing with mRNA length and running with the following parameters: method=Wallenius, repcnt=2 000, use_genes_without_cat=F. Categories were considered to be over-represented if the associated *P*-value was <0.05 after Benjamini–Hochberg correction.

### sRNA-Seq

sRNAs were cloned from 10 µg total RNA (from the same tissue as used for RNA-Seq, for two wild-types and two pairs of *sulf* and *sulf*/+) using the Illumina TruSeq Small RNA cloning kit and libraries were indexed during the PCR step (12 cycles) according to the manufacturer’s protocol. Gel size-selected, pooled libraries were sequenced on a HiSeq 2000 50SE. Sequences were trimmed and filtered with Trim Galore! (with the adapter parameter -a TGGAATTCTCGGGTGCCAAGG) and 14–20 million reads per library were mapped without mismatches and clustered on Heinz genome SL2.50 using the ShortStack software v2.1.0 (Axtell, 2013; Supplementary Table S2). sRNA counts on the defined loci were analysed with DESeq2 v1.8.1. Uniquely mapping reads on DMR1 and DMR2 were normalized with edgeR’s implementation of TMM size factors, on all sRNAs present in all libraries and with at least 10 total counts (Robinson *et al.*, 2009), and a Poisson regression was applied to the normalized counts (generalized linear model in R, with the genotype variable taking values wt, *sulf*/+, and *sulf*).

### Methylome analysis

DNA was extracted from 100mg of leaf tissue (from the same sampling as for RNA-Seq and sRNA-Seq, for two wild-types and two *sulf*) using the Puregene kit (QIAGEN). Bisulﬁte library preparation was performed with a custom protocol similar to Urich *et al.* (2015).1.2 µg DNA was sonicated on a Covaris E220 to a target size of 400bp and puriﬁed on XP beads (Ampure, ratio 1.8). DNA was end-repaired and A-tailed using T4 DNA polymerase and Klenow Fragment (NEB) and puriﬁed again using XP beads (ratio 1.8×). Methylated Illumina Y-shaped adapters for paired-end sequencing were ligated using Quick-Stick Ligase (Bioline). 450ng of puriﬁed (ratio 1.8×), adapter-ligated DNA was bisulﬁte-converted using the EZ DNA Methylation-Gold Kit (Zymo Research) according to the manufacturer’s instructions. DNA was barcoded using 12 cycles of PCR ampliﬁcation with KAPA HiFi HotStart Uracil+Ready Mix (Kapa Biosystems) with PE1.0 and custom index primers (courtesy of the Sanger Institute). Pooled libraries were sequenced to a depth of about 5× on a HiSeq 2500 125PE. Sequences were trimmed and ﬁltered with Trim Galore! (default parameters), then mapped on Heinz genome SL2.50 using Bismark v0.14.3 (Krueger and Andrews, 2011) (first in paired-end mode with options --score-min L,0,-0.2 -p 4 --reorder --ignore-quals --no-mixed --no-discordant -X 1500 --unmapped --ambiguous, then unmapped read1 was mapped in single-end mode with the same quality parameter -N 1, Supplementary Table S3). Reads were deduplicated with bismark_deduplicate and methylation calls were extracted using Bismark methylation_extractor (with options -r2 2 for paired end reads). Methylated and unmethylated counts for cytosines of both strands were pooled into contiguous 200bp bins and separated by context (CG, CHG, and CHH) with a custom python script. Bins with fewer than 10 counts were excluded from the analysis. Bins are considered differentially methylated if the maximum *P*-value of the two chi-square tests (wt1 versus *sulf* 1, wt2 versus *sulf* 2) is <0.01. Analysis of methylation by McrBC was performed as previously described by Bond and Baulcombe (2015). For Sanger bisulfite sequencing, 450ng of DNA was bisulfite-converted, amplified with primers specific to the region of interest, A-tailed, and cloned into pGEM-T easy (Promega) following protocols similar to the library preparation. Sequences aligned with MUSCLE were then analysed with CyMATE (Hetzl *et al.*, 2007).

### VIGS

DMR1a (606bp) and DMR1b (562bp) genomic inserts were cloned into the binary TRV RNA2 vector using the *Kpn*I and *Xho*I restriction sites of the multiple cloning site as described previously (Liu *et al.*, 2002; Bond and Baulcombe, 2015). Cotyledons of tomato seedlings were agro-infiltrated 10 d after sowing with a 1:1 mixture of *Agrobacterium tumefaciens* (strain GV3101:pMP90+pSOUP) carrying TRV RNA1 and RNA2 at OD_600_=1.5. Symptoms of *SLTAB2* silencing were visible from 2 weeks post-infection.

### Auxin quantification

Endogenous levels of free IAA were detected by LC-MS/MS method as described in Novák *et al.* (2012). Briefly, 10–20mg fresh tissue of the control and mutant lines were collected, extracted in ice-cold 50mM sodium phosphate buffer (pH 7) and puriﬁed by SPE on hydrophilic–lipophilic balance reversed-phase sorbent columns (Oasis HLB, 1 cc/30mg, Waters). To each extract, 5 pmol of ^13^C_6_-IAA were added as internal standards to validate the quantiﬁcation. Puriﬁed samples were analysed by the LC-MS/MS system consisting of an ACQUITY UPLC System (Waters, Milford, MA, USA) and Xevo TQ-S (Waters) triple quadrupole mass spectrometer. Quantiﬁcation was obtained using a multiple reaction monitoring (MRM) mode of selected precursor ions and the appropriate product ion.

### Oligonucleotides

Please refer to Supplementary Table S4.

### Accession codes

All sequencing data have been deposited in the Sequence Read Archive under the BioProject SRP066362.

## Results

### Pericentromeric *SLTAB2* is strongly down-regulated in *sulfurea*

**Fig. 1.**
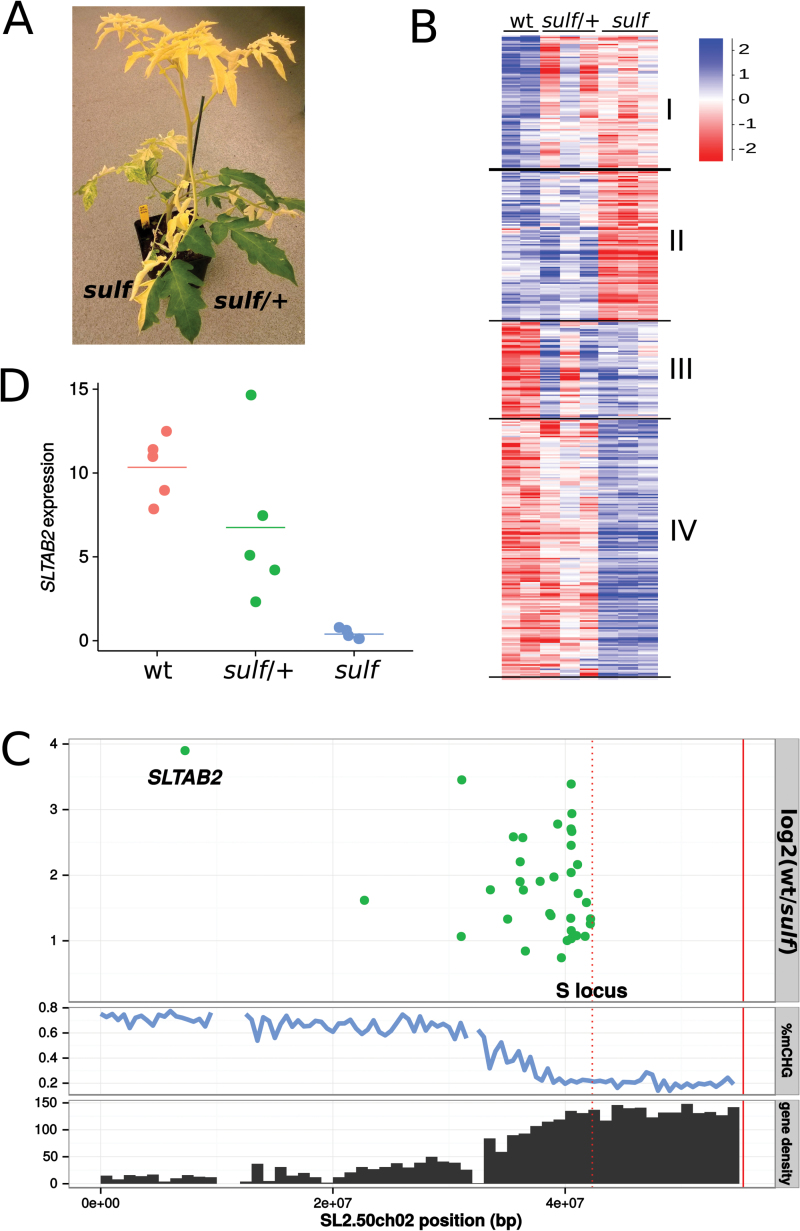
(A) Fully paramutated yellow *sulf* tissue and non-paramutated green *sulf*/+ tissue on a plant grown from a heterozygous seed. (B) Hierarchical clustering of 2 237 differentially expressed genes between wt and *sulf*. With a correlation coefficient of 0.7, 2 223 genes fall into four broad categories: down-regulated in *sulf* and *sulf*/+ (I), down-regulated in *sulf* only (II), up-regulated in *sulf* and *sulf*/+ (III), and up-regulated in *sulf* only (IV). Values are log_2_-transformed library-normalized/median-normalized counts. (C) Down-regulated genes in *sulf* on chromosome 2. Log_2_ fold-change wt/*sulf* of signiﬁcantly down-regulated genes (adjusted *P* <0.05). Percentage CHG methylation and gene density (genes/Mb) are plotted along chromosome 2 to show the distribution of heterochromatin. The *S* locus (red dotted line) marks the right-most border for the possible location of *SULFUREA* (Hagemann and Snoad, 1971). The end of the chromosome is marked by a solid red line. (D) *SLTAB2* expression in wt, *sulf/*+ (green) and *sulf* (yellow) leaves. Horizontal bar shows the mean of five biological replicates. 26-fold reduction in *sulf* compared with wt (*P*-value=0.0002324, two–tailed *t* test), while the difference between wt and *sulf*/+ is not signiﬁcant (*P*-value=0.1771).

The tomato lines in this study had either unsilenced *SULF* loci (wild type) or they were the progeny of a cross between the wild type and a plant with sectors with silent *sulf*. Some of the F1 plants were wild type and fully green or, like the sectored parent, they had green and chlorotic sectors consistent with a heterozygous *sulf*/+ epigenotype with paramutation. The chlorotic sectors would have had the homozygous *sulf*/*sulf* epigenotype (referred to as *sulf*) and the non-paramutated green sectors would be *sulf*/+ (Fig. 1A).

Based on the understanding of paramutation in maize, the *SULF* locus would be suppressed in *sulf* (paramutated yellow sectors), partially silent in *sulf*/+ (non-paramutated green sectors) and fully expressed in the wild type (wt). It would also be located in the pericentromeric heterochromatin of chromosome 2 upstream of the euchromatic *S* locus (Hagemann and Snoad, 1971). To find loci with these characteristics, we analysed transcripts of wild-type, non-paramutated *sulf*/+, and paramutated *sulf* leaves using mRNA-seq. We identiﬁed 2 237 differentially expressed genes between *sulf* and wt (*P* <0.05) that clustered into four main categories with distinct Gene Ontology enrichments (Fig. 1B; Table 1). Consistent with a decrease in photosystem I and the quantity of pigment in *sulf* leaves (Ehlert *et al.*, 2008), many photosynthesis-related genes were down-regulated (Table 1, class II). The down-regulation of photosystem I was also detectable in the non-paramutated heterozygous *sulf*/+ leaves (Table 1, class I). Genes associated with various stress responses were up-regulated in *sulf* (Table 1, class IV) and were probably a secondary consequence of the *sulf* phenotype.

**Table 1.** Enriched gene ontology terms in the 4 hierarchical clusters

Down-regulated in *sulf* and *sulf*/+ (I, 467 genes)	Down-regulated in *sulf* (II, 524 genes)	Up-regulated in *sulf* and *sulf*/+ (III, 339 genes)	Up-regulated in *sulf* (IV, 893 genes)
Sucrose metabolic process	Photosystem II	Tyramine N-feruloyltransferase activity	Nucleolus
Starch metabolic process	Photosynthesis, light harvesting	Plasma membrane part	Ribosome biogenesis
Microtubule	Chlorophyll binding	Killing of cells of other organism	Mitochondrion
Microtubule-based movement	Protein-chromophore linkage	*N*-acetyltransferase activity	Translation
Endomembrane system	Photosystem I		Unfolded protein binding
Plasma membrane	Chloroplast thylakoid membrane		Ribosome
Microtubule motor activity	Integral component of membrane		Response to heat
Cellulase activity	Light-harvesting complex		RNA binding
Protein-chromophore linkage	Metal ion binding		Arsenate reductase (glutaredoxin) activity
Mannan synthase activity	Membrane		Structural constituent of ribosome
Photosystem I	Plastoglobule		Chloroplast
Metal ion transport	Sterol biosynthetic process		snoRNA binding
Chlorophyll binding	Photosystem II antenna complex		Response to stress
Carbohydrate metabolic process	Water transport		RNA processing
	Response to red light		Protein folding
	Response to blue light		Plastid chromosome
	Response to water deprivation		Protein disulfide oxidoreductase activity
	Water channel activity		DNA-directed RNA polymerase activity
	Plasma membrane light-harvesting complex		Binding
	Electron carrier activity		Purine nucleobase metabolic process
	Chlorophyll biosynthetic process		Cytosolic large ribosomal subunit
	Osmosensor activity		Protein refolding
	Non-photochemical quenching		Cell redox homeostasis
	Transferase activity, transferring hexosyl groups		Nucleoid
			ATP-dependent helicase activity
			Pyrimidine nucleobase metabolic process
			Translation elongation factor activity
			Protein processing
			Plastid organization
			Endonuclease activity
			Cobalt ion binding

Of these differentially expressed genes, 36 were both down-regulated in *sulf* and located upstream of the *S* locus on chromosome 2. Among these candidates for *SULF*, Solyc02g005200 particularly stood out as being the most repressed in *sulf* (15-fold reduction) and at the predicted map position of *SULF* (29 cM from *S* locus, when the centromere-*S* distance is 30 cM). The other candidates mapped to the euchromatin or the transition zone between heterochromatin and euchromatin (Fig. 1C; Supplementary Table S5). Further qPCR analysis conﬁrmed the strong down-regulation of Solyc02g005200 in *sulf* (26-fold) and revealed variable levels in *sulf*/+, compatible with mono-allelic expression (Fig. 1D). Solyc02g005200 is the orthologue of *Arabidopsis thaliana ATAB2* that is likely involved in the translation of mRNAs for both photosystems (Barneche *et al.*, 2006) and we refer to it as *SLTAB2*.

**Fig. 2.**
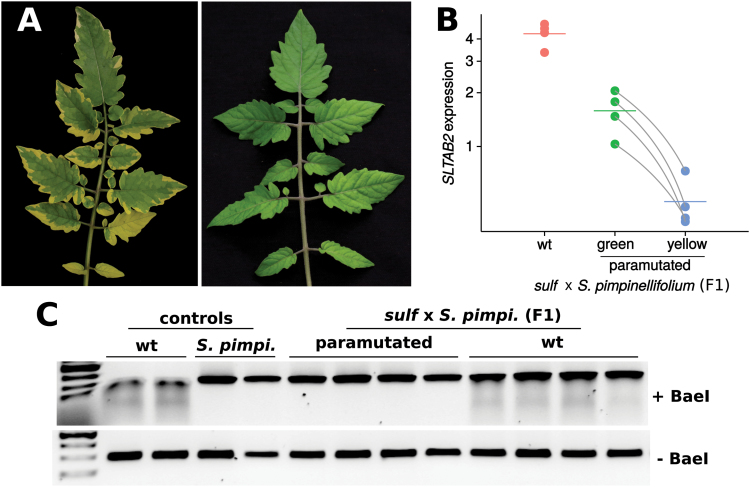
*SLTAB2* paramutation in the cross between a heterozygous *sulf*/+ and *S. pimpinellifolium*. (A) Phenotypes of a paramutated (left) and wild-type leaf (right) in the F1. (B) *SLTAB2* expression in the F1. Paramutated plants showed reduced expression in the green sectors compared with wt plants (*P*=7.8e-4, two-tailed *t* test), and even further reduction in the chlorotic sectors (*P*=6.6e-3, paired *t* test between green and yellow samples). Data are plotted on a log_2_ scale, the mean is represented by a horizontal bar. Paired data points for paramutated plants (green and yellow sectors) are joined by grey lines. (C) Only the *S. pimpinellifolium SLTAB2* allele is expressed in paramutated F1 plants. The *S. lycopersicum* allele is sensitive to digestion by the BaeI restriction enzyme, resulting in a smear. Two SNPs in the *S. pimpinellifolium SLTAB2* allele make it resistant to BaeI treatment. F1s expressing the *S. lycopersicum* allele show a smear, whereas F1s in which the *S. lycopersicum* allele is silent do not.

To confirm the heritability of the *SLTAB2* silent epiallele, we analysed *SLTAB2* in the F1 progeny of chlorotic *sulf*/+ (*S. lycopersicum* cv. Lukullus) crossed with *S. pimpinellifolium*. Out of 22 F1 plants, 8 displayed a paramutated phenotype with yellowing of parts of the leaves (Fig. 2A). The expression of *SLTAB2* in these chlorotic plants was reduced by about half in their green sectors compared to wild-type plants, and 9-fold in their yellow sectors (Fig. 2B). Furthermore, in a PCR test that differentiated the polymorphic alleles from the two parents, we only detected expression from *S. pimpinellifolium* (Fig. 2C). These data are consistent with *SLTAB2* being *SULFUREA*: in the green tissue the *S. pimpinellifolium* allele would have been expressed (but not the silent allele from the *sulf*/+ *S. lycopersicum* parent), and in the chlorotic tissue, it would have been paramutated. In addition, by confirming the heritable silencing of the *S. lycopersicum* allele in the chlorotic plants, these data confirm that *SLTAB2* silencing is not merely a consequence of the *sulf* phenotype.

**Fig. 3.**
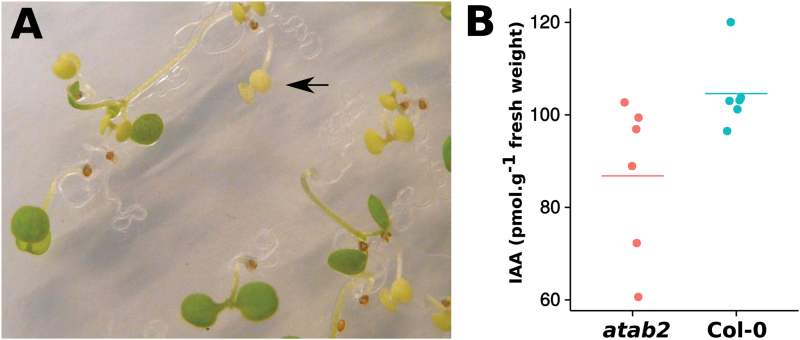
*Arabidopsis atab2* mutants resemble tomato *sulfurea*. (A) Segregating *atab2* mutation in seedlings. Homozygous *atab2* mutants (arrow) are chlorotic and seedling-lethal on heterotrophic medium. (B) Decreased auxin in *atab2* (*P*-value=0.02497, Kruskal-Wallis rank sum test).

Furthermore, consistent with equivalence of *SULF* and *SLTAB2*, the *Arabidopsis* T-DNA knockout of *ATAB2* is seedling-lethal in heterotrophic conditions and deﬁcient in green pigment (Barneche *et al.*, 2006; Fig. 3A). This mutant also has less auxin than the wild type (Fig. 3B). These three phenotypes all resemble *sulf*.

### Paramutation is associated with changes in *SLTAB2* promoter DNA methylation

**Fig. 4.**
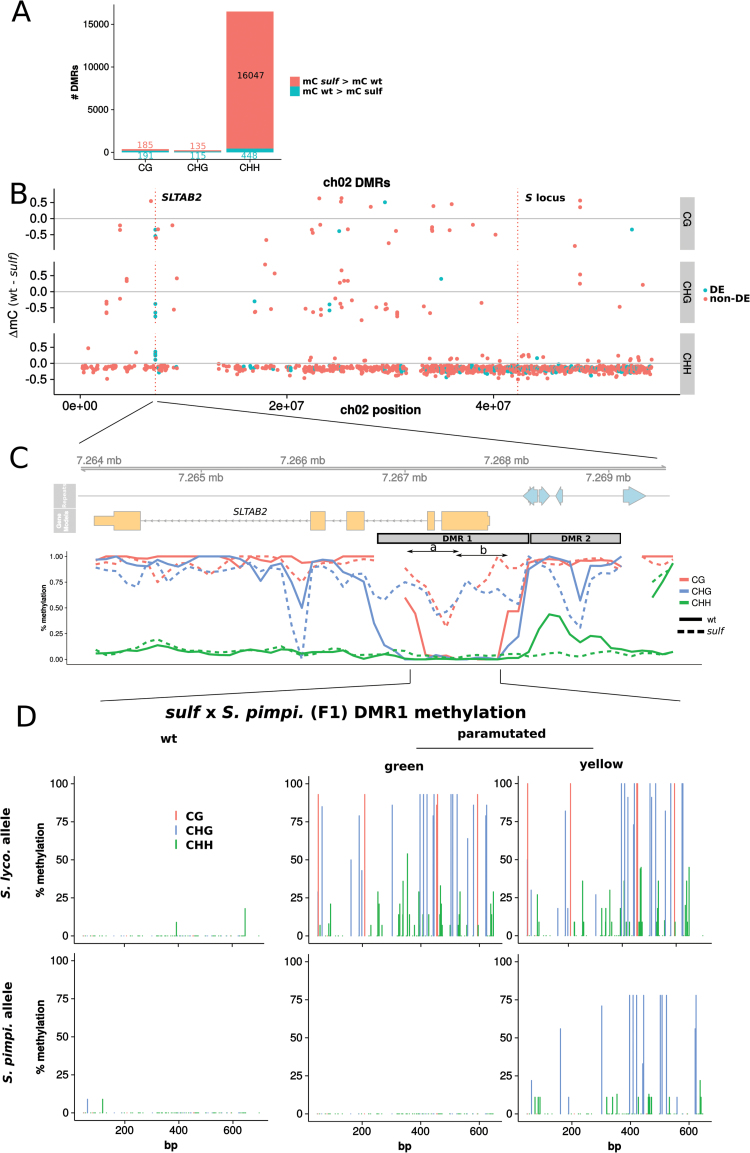
DMRs between wild-type and *sulfurea*. (A) Hypo- and hyper-DMRs between wild-type and *sulfurea*. DMRs in the CHH context are the most abundant (1% of tested bins), and heavily biased for hypermethylation in *sulf*. (B) Methylation difference (wt–*sulf*) of chromosome 2 DMRs. Negative values indicate hypermethylation in *sulfurea*, positive values hypomethylation. DMRs whose downstream gene is differentially expressed (DE) between wild-type and *sulfurea* are coloured in blue, while DMRs whose downstream gene is not differentially expressed (non-DE) are coloured red. (C) Methylation over *SLTAB2* promoter, plotted in 200bp sliding windows (step of 100bp). Repeats are plotted as blue arrows. (D) Wild-type *sulf*×*S. pimpinellifolium* F1s bear no methylation on either the *S. lycopersicum* or *S. pimpinellifolium* alleles at DMR1 (region 7 267 172 to 7 267 898 encompassing the *S. pimpinellifolium* SNPs, 11 clones per allele). In the green sectors of paramutated F1s only the *S. lycopersicum* allele is methylated (14 clones, 7 clones for *S. pimpinellifolium* allele), whereas in the yellow sectors methylation is found on both alleles (11 and 9 clones).

We predicted, based on the analysis of the maize *b1* gene (Stam *et al.*, 2002), that the DNA of the paramutagenic *sulf* would be hypermethylated. After a genome-wide analysis of differentially methylated regions (DMRs) between wt and *sulf*, we looked for candidate loci in the appropriate region of chromosome 2 and adjacent to genes that were differentially expressed. Genome-wide there were thousands of such DMRs in the CHH context and hundreds in the CG and CHG contexts, with the CHH DMRs being predominantly hypermethylated in *sulf* whereas CG and CHG DMRs were evenly split between hyper- and hypo-DMRs (Fig. 4A). On chromosome 2 there were several differentially expressed genes with adjacent CHH DMRs but only the *SLTAB2* locus had strong DMRs in all cytosine contexts (Fig. 4B).

Closer inspection revealed that there are two adjacent DMRs in the immediate promoter of *SLTAB2*, DMR1 and DMR2 (Fig. 4C; Table 2). DMR2 overlaps annotated repeats directly upstream of *SLTAB2* and was hypomethylated in *sulf* in the CHG and CHH contexts (78% and 4% methylation, respectively, compared with 93% and 27% in the wild type). DMR1, by contrast, overlaps the transcriptional start site of *SLTAB2* from 300bp upstream, encompassing the first two exons and introns, and it was hypermethylated in *sulf* in all contexts (73% mCG, 62% mCHG, and 4% mCHH compared with 24%, 4%, and 0.7%, respectively, in wt). This hypermethylation of the transcriptional start site of *SLTAB2* is consistent with a decrease in transcription in *sulf* and it further strengthens the case that *SLTAB2* is *SULF*.

**Table 2.** *SLTAB2* DMR methylation Percentage methylation from two replicates. *P*-values from a logistic regression analysis on raw methylated and unmethylated cytosine counts.

DMR	Context	wt	*sulf*	*P*-value
DMR1	CG	21.4; 27.3	71.4; 76.8	<2×10^–16^
	CHG	2.3; 5.8	61.9; 62.4	<2×10^–16^
	CHH	0.5; 0.8	3.8; 4.8	1.12×10^–7^
DMR2	CG	95.6; 96.4	96.6; 93.7	0.557
	CHG	94.4; 90.9	78.0; 78.4	7.34×10^–4^
	CHH	25.6; 29.3	5.5; 3.6	<2×10^–16^

Additional evidence for the relevance of DMR1 methylation for paramutation is our finding that the silencing of the *S. pimpinellifolium* allele in the chlorotic F1 (*sulf*/+×*S. pimpinellifolium*) coincided with a hypermethylation of this region (Fig. 4D). The silent *S. lycopersicum* allele was methylated in all parts of the paramutated plants, whereas the *S. pimpinellifolium* allele was unmethylated in green sectors and methylated in yellow sectors.

**Fig. 5.**
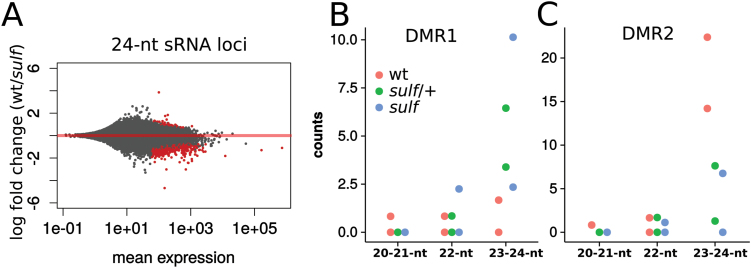
sRNAs in *sulfurea*. (A) MA plot of 24-nt sRNA loci in wt and *sulf*. Of the differential sRNA loci between wt and *sulf* (in red, adjusted *P* <0.05), 498 had more abundant sRNAs in *sulf*, while only 48 had more abundant sRNAs in the wild type. (B) sRNA counts on DMR1 in the wild type, heterozygous *sulf*/+, and homozygous paramutated *sulf* leaves. 23–24-nt sRNAs are rare but more abundant in *sulf* (*P*=0.0146, Poisson regression). (C) sRNA counts on DMR2. 23–24-nt siRNAs are reduced in *sulf* (*P*=5.48*e* − 05, Poisson regression). Counts in (B) and (C) are for normalized, uniquely mapping reads.

We also predicted, based on the maize paramutation examples, that *sulf* would correlate with 24-nt siRNAs. At a genome-wide scale, there was a distinct increase in 23–24-nt siRNAs in *sulf* compared with the wild type (Fig. 5A), in line with the pattern of CHH hypermethylation. At DMR1 of *SLTAB2*, the 23–24-nt siRNAs were more abundant in paramutated *sulf* (Fig. 5B) than in the wild type whereas at DMR2 the 23–24-nt siRNAs were less abundant than in the wild type (Fig. 5C).

### VIGS of *SLTAB2* phenocopies *sulf*

**Fig. 6.**
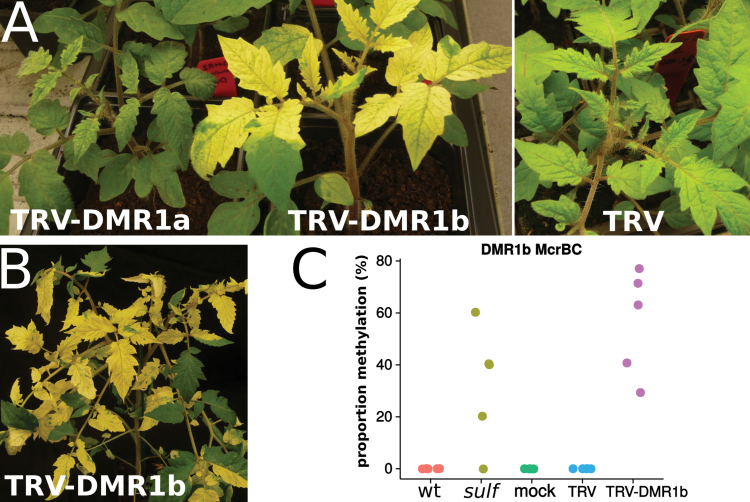
VIGS of *SLTAB2* DMR1. (A) At 3 weeks post-infection, plants infected with TRV-DMR1b displayed large chlorotic sectors. Between 13 and 16 plants were infected for each condition. (B) These *sulfurea*-like sectors remained in later leaves at 2 months after infection. (C) Methylation of DMR1 is detectable in chlorotic sectors. The proportion of methylation is calculated from the ratio of amplicons in the McrBC-digested versus undigested samples (as determined by qPCR). If all alleles are highly methylated (100% methylation), they will be digested by McrBC and no amplification will occur during qPCR.

To test the involvement of *SLTAB2* in *sulf* paramutation further, we used virus-induced gene silencing (VIGS). VIGS, when targeted to transcribed sequences, leads to knock-down of mRNA levels by post-transcriptional gene silencing but, when targeted at DNA sequences [e.g. the *FWA* promoter in *A. thaliana* (Bond and Baulcombe, 2015)], it can initiate heritable DNA methylation and transcriptional gene silencing. We cloned two segments (a and b, Fig. 4C) of DMR1 into tobacco rattle virus (TRV) RNA2 and inoculated them with TRV RNA1 to wild-type tomato. While infection with TRV-DMR1a caused only mild variegation of the leaves, infection with TRV-DMR1b caused almost all plants to develop large *sulf*-like chlorotic sectors (Fig. 6A). The similarity of this VIGS phenotype to leaves of *sulf* is further evidence that *SLTAB2* and *SULF* are equivalent.

DNA methylation analysis of chlorotic sectors by McrBC suggested that there is an epigenetic component to the silencing of *SLTAB2* by TRV-DMR1b: the targeted DNA was as strongly methylated as in *sulf* samples (Fig. 6C). The involvement of epigenetics is further supported by the lasting VIGS phenotype several months post-inoculation (Fig. 6B) during which time the level of the virus vector decreased. From these data we conclude that the DMR1b region of *SLTAB2* has the predicted characteristics of the paramutagenic component of *sulf* because it is susceptible to epigenetic modification.

## Discussion

In this paper we present several lines of evidence that *SLTAB2* is *SULF*: it maps closer to *SULF* than any other genes with the predicted pattern of mRNA accumulation in *sulf*/+ and *sulf* tissue (Fig. 1B); it encodes a protein required for photosystem I production that explains the chlorotic phenotype; the orthologous *atab2* mutation has the same chlorosis and auxin-deficient phenotype as *sulf* (Fig. 3); and there is a definite DNA methylation mark at the silent *SLTAB2* allele in *sulf* that is inherited from *sulf*/+ both in selfed and outcrossed progeny (Fig. 2). This epigenetic mark is transferred to the previously active allele in F1 progeny (Fig. 4D) and is associated with 24-nt siRNAs (Fig. 5). The final evidence for the equivalence of *SLTAB2* and *SULF* is from the finding that VIGS targeted to the *SLTAB2* DMR can recapitulate both the physiological and epigenetic features of *sulf* (Fig. 6).

We envision that paramutation occurs when methylation of DMR1 DNA by paramutagenic siRNAs starts a positive feedback loop in which Pol IV is recruited to the silent locus by SHH1 (Law *et al.*, 2013). The recruited Pol IV (Blevins *et al.*, 2015; Zhai *et al.*, 2015) would transcribe siRNAs that would mediate maintenance of the silent state of *SLTAB2* and its transfer to paramutable alleles.

*SLTAB2* had been ruled out previously as *SULF* because it is expressed at detectable levels in *sulf* and it was thought that, unlike tomato, the mutation of the *Arabidopsis* orthologue could be rescued on sucrose (Ehlert *et al.*, 2008). An alternative candidate for *SULF* was implicated in the tryptophan-independent pathway of auxin biosynthesis. With our new data, however, we show that the previous exclusion of *SLTAB2* was not valid because targeted suppression by VIGS or mutation of this gene produces an accurate phenocopy of the *sulf* phenotype including, in the *atab2* mutant, an auxin defect and seedling lethality.

A likely explanation for the *sulf* phenotype based on silencing of *SLTAB2* invokes the failure to translate the psaB mRNA as described for the orthologous mutations *ATAB2* in *Arabidopsis* and *TAB2* in *Chlamydomonas* (Dauvillée *et al.*, 2003; Barneche *et al.*, 2006). The PsaB protein is the reaction centre protein of photosystem I and, in its absence, the thylakoid membranes would fail to form, pigments would not accumulate at the normal levels, and the leaves would be chlorotic. An auxin defect of *sulf* is a likely consequence of the PsaB defect, as observed in the *atab2* mutant (Fig. 3). In addition, the genome-wide hypermethylation in the CHH context in *sulf* is reminiscent of transient hypermethylation in response to stress, already described in phosphate-starved rice (Secco *et al.*, 2015) and virus-infected *Arabidopsis* (Bond and Baulcombe, 2015).

Several cases of paramutation have been tied to the hypermethylation of regulatory tandem repeats (Stam *et al.*, 2002; Chandler and Stam, 2004). By contrast, the *sulfurea* paramutation is correlated with increased DNA methylation at the transcriptional start site (DMR1) where there is no repeated DNA (Fig. 4). There are annotated LTR fragments at DMR2 that is directly adjacent to DMR1 but, unlike the classic systems, this region of the paramutagenic *sulf* allele showed a reduction in sRNAs and hypomethylation. Hypomethylation of DMR2 may be a consequence of a different chromatin state of the silenced allele and, although its repeats may not be directly involved in the silencing of *SLTAB2*, they and the largely heterochromatic region in which *SLTAB2* is embedded may contribute to its paramutability, poising it for silencing.

The opportunity to study paramutation via *SLTAB*/*SULF* in tomato has several advantages over the various maize systems that have been most informative until now. First we have a VIGS system so that establishment of the epigenetic mark can be tracked directly in tomato mutants that are defective for components of the RNA silencing pathways. We will also be able to use VIGS on *sulf*/+ plants to test the role of various tomato genes in the establishment and maintenance of paramutagenicity and paramutability.

A second beneﬁt of the *sulf* system is the possibility of studying the transfer of the epigenetic mark in vegetative tissue. With the well-studied maize paramutation systems this transfer is likely to occur early in embryo development and is not readily accessible to molecular analysis, whereas, in tomato, it will be taking place in or close to vegetative meristems. It will still not be easy to access the cells in which the allelic transfer is taking place but we may be able to use the DMR1-specific siRNAs as markers of the transfer process. These RNAs are rare in total plant extracts (Fig. 5) but they may be more abundant at the primary sites of paramutation. Having identiﬁed the *SULF* gene, we should also be able to complement the physiological consequences by providing a transgene without the target DNA of paramutation so that we can grow plants with a *sulf*/*sulf* epigenotype.

These various experimental tools will allow us to explore the differences of the tomato and maize paramutation systems. For example, the apparent target of paramutation in *sulf* has no tandem repeats and is close to the transcriptional start whereas, in maize, at the *B* locus, they are essential and separated from the transcribed region by 100kb. Answers to these and other questions will allow us to explore the frequency of paramutation-like events in plant breeding and evolution. Several recent ﬁndings indicate that such events are not restricted to the few well-characterized examples of paramutation in maize and other species (Regulski *et al.*, 2013; Greaves *et al.*, 2014). They may be frequent and have an effect on transgressive and heterotic phenotypes.

## Supplementary data

Supplementary data are available at *JXB* online.

Table S1. Summary of RNA-Seq alignments.

Table S2. Summary of sRNA-Seq alignments.

Table S3. Summary of whole-genome bisulfite sequencing alignments.

Table S4. List of primers.

Table S5. Summary of down-regulated genes in the genetically defined region of *SULFUREA.*

Supplementary Data
