# Supplementary material for: SLTAB2 is the paramutated SULFUREA locus in tomato
Source: J Exp Bot. 2016 Mar 8;67(9):2655–64. doi: 10.1093/jxb/erw096 (PMC4861014; doi:10.1093/jxb/erw096)
Supplement: Supplementary Data [file supp_67_9_2655__index.html]

 SLTAB2 is the paramutated SULFUREA locus in tomato — SLTAB2 is the paramutated SULFUREA locus in tomato — Supplementary Data 

# *SLTAB2* is the paramutated *SULFUREA* locus in tomato

## Supplementary Data

Data files

- supplementary\_tables\_S1\_S5.pdf - Supplementary Data
