# Supplementary material for: SLTAB2 is the paramutated SULFUREA locus in tomato
Source: J Exp Bot. 2016 Mar 8;67(9):2655–64. doi: 10.1093/jxb/erw096 (PMC4861014; doi:10.1093/jxb/erw096)
Supplement: Supplementary Data [file supp_erw096_supplementary_tables_S1_S5.pdf]

Supplementary table 1: summary of RNA-Seq alignments.

library	alignment rate	# read pairs mapped
wt 1	73.2%	22843633
wt 2	67.8%	29047268
sulf 1	68.1%	12503876
sulf/+ 1	66.4%	11706233
sulf 2	69.5%	12336371
sulf/+ 2	69.4%	12165523
sulf 3	71.8%	17220956
sulf/+ 3	67.1%	10809351

Supplementary table 2: summary of sRNA-Seq alignments.

library	# reads mapped
wt 1	15706743
wt 2	18315830
sulf 1	19570113
sulf/+ 1	18922877
sulf 2	13778995
sulf/+ 2	18009323

Supplementary table 3: summary of whole-genome bisulfite sequencing (WGBS) alignments, after deduplication.

library	# mapped pairs	# read1 mapped in SE	coverage
wt 1	12786917	7138458	4.8x
wt 2	14005678	8596462	5.4x
sulf 1	14778533	6474490	5.3x
sulf 2	18766251	6211632	6.4x

Supplementary table 4: Oligonucleotides used in the present study.

Experiment	Name	Sequence (5'-3')
qPCR	sltab2 fw	CCTGAGAGCTTATCTGAGTGGG
	sltab2 rv	ACAAGTGATAAGCACCGTTTGC
	tip41 fw	ATGGAGTTTTTGAGTCTTCTGC
	tip41 rv	GCTGCGTTTCTGGCTTAG
	expr fw	GCTAAGAACGCTGGACCTAAT
	expr rv	TGGGTGTGCCTTTCTGAATG
VIGS cloning	DMR1a fw	CCGGTACCGTCGTGGGTGCTCACTTTCTTT
	DMR1a rv	CCCTCGAGAGCTTATCTGAGTGGGAATTGGA
	DMR1b fw	CCGGTACCTGAAGAGAGAGGGAATCATCACA

	DMR1b rv	CCCTCGAGTGTTGAATTGGGCAAAGGACAA
McrBC assay	sltab2M fw	TCTCCAACATTTGAGGGTCGTG
	sltab2M rv	CCTGAGAGCTTATCTGAGTGGG
Bisulfite	DMR1 fw	GGATTGAAATGTTGTATATATAGGG
	DMR1 rv	CTTCTARTCTCCCTTATCTTTTAAA

Supplementary table 5: summary of down-regulated genes in the genetically defined region of *SULFUREA*. Chromosome position (start and end), basal level of expression (baseMean), log2 fold-change between wt and sulf, adjusted p-value (padj). CG, CHG and CHH DMRs indicate whether there was a differentially methylated region in the relevant context assigned to the gene (each DMR was assigned to the nearest gene). For the CHH DMRs, their distance to the gene is also reported.

**gene**	**chromosome**	**start**	**end**	**baseMean**	**log2FC(wt/*sulf*)**	**padj**	**CG DMR**	**CHG DMR**	**CHH DMR**	**distance (CHH)**	**annotation**
Solyc02g005200.2	SL2.50ch02	7263948	7267830	183.41	4.48	3.52E-013	hyper	hyper	hyper/hypo	0	SLTAB2, PsaB translation factor
Solyc02g021440.2	SL2.50ch02	22714574	22721523	236.35	1.58	3.56E-005	-	-	hyper	<1kb	serine threonine kinase
Solyc02g037530.2	SL2.50ch02	31048794	31059050	236.81	1.22	2.25E-003	-	-	hyper	1-2kb	auxin response factor 8B
Solyc02g037550.2	SL2.50ch02	31094995	31098669	142.74	3.34	2.28E-003	-	-	hyper	3-4kb	Auxin efflux carrier family protein
Solyc02g061990.2	SL2.50ch02	33560595	33561944	24.70	3.29	3.16E-003	-	-	hyper	5-6kb	Basic leucine zipper transcription factor
Solyc02g062330.1	SL2.50ch02	33942894	33947887	16.65	2.39	8.33E-003	-	-	hyper	<1kb	Kinesin like protein
Solyc02g063030.2	SL2.50ch02	35047619	35051492	1622.94	1.35	6.52E-004	-	-	hyper	<1kb	LuxR family transcriptional regulator
Solyc02g063450.2	SL2.50ch02	35542967	35548538	716.36	3.03	2.79E-005	-	-	-		Hypothetical YFW family protein 5
Solyc02g064720.2	SL2.50ch02	35825774	35829784	126.50	1.13	3.25E-002	-	-	-		Phototropic-responsive NPH3 family protein
Solyc02g064990.2	SL2.50ch02	36118886	36121206	24.76	2.39	1.41E-003	-	-	hyper	3-4kb	remorin, plant-specific marker for membrane
Solyc02g065000.1	SL2.50ch02	36129001	36129522	237.91	2.50	2.12E-005	-	-	hyper	<1kb	Calmodulin-like protein
Solyc02g065190.2	SL2.50ch02	36351922	36354372	40.03	4.57	5.92E-007	-	-	hyper	1-2kb	Cytochrome P450
Solyc02g065230.2	SL2.50ch02	36403136	36404871	64.18	2.17	4.88E-003	-	-	hyper	2-3kb	Cytochrome P450
Solyc02g065400.2	SL2.50ch02	36573934	36576914	43311.91	0.87	1.06E-002	-	-	-		Oxygen-evolving enhancer protein 1 of photosystem II
Solyc02g065610.2	SL2.50ch02	36775106	36779309	311.99	1.02	2.02E-002	-	-	hyper	0	Leaf senescence protein-like
Solyc02g067690.2	SL2.50ch02	37817307	37819014	61.82	1.66	9.46E-003	-	-	hyper	<1kb	Glucosyltransferase-like protein
Solyc02g067730.1	SL2.50ch02	37846237	37848096	12.94	3.93	1.57E-004	-	-	hyper	2-3kb	RNA exonuclease 4
Solyc02g068740.2	SL2.50ch02	38671673	38675621	222.70	1.47	1.09E-004	-	-	hyper	1-2kb	Glycine cleavage system H protein 1
Solyc02g068900.2	SL2.50ch02	38779435	38783709	499.63	1.59	2.01E-002	-	-	-		Glutathione transferase
Solyc02g069060.2	SL2.50ch02	39033490	39034511	55.59	2.33	1.09E-002	-	-	-		Phloem lectin
Solyc02g069450.2	SL2.50ch02	39337837	39341402	47270.82	1.40	1.72E-003	-	-	-		Photosystem I reaction center subunit III
Solyc02g069460.2	SL2.50ch02	39339989	39341652	37771.88	1.45	1.13E-003	-	-	-		Photosystem I reaction center subunit III
Solyc02g069490.2	SL2.50ch02	39357631	39359749	14018.31	2.91	1.51E-015	-	-	-		FAD linked oxidase domain protein
Solyc02g069860.2	SL2.50ch02	39676145	39682167	2174.91	0.70	1.44E-002	-	-	-		Nuclear transcription factor Y subunit A-7
Solyc02g070430.2	SL2.50ch02	40156961	40159000	11.06	2.85	1.86E-002	-	-	hyper	<1kb	Gibberellin 2-oxidase 1
Solyc02g070440.2	SL2.50ch02	40174602	40181876	441.00	1.30	1.44E-004	-	-	hyper	5-6kb	Katanin p60 ATPase-containing subunit A
Solyc02g070530.2	SL2.50ch02	40261306	40264437	175.13	0.99	1.76E-002	-	-	hyper	3-4kb	Potassium channel
Solyc02g070880.1	SL2.50ch02	40480871	40481611	66.47	1.51	8.33E-003	-	-	hyper	2-3kb	BHLH1 transcription factor
Solyc02g070950.1	SL2.50ch02	40505514	40506311	8312.01	2.79	1.93E-008	-	-	-		Chlorophyll a/b binding protein
Solyc02g070970.1	SL2.50ch02	40512599	40513396	8396.36	2.15	1.19E-006	-	-	-		Chlorophyll a/b binding protein
Solyc02g070980.1	SL2.50ch02	40515041	40515838	5900.04	2.84	4.50E-007	-	-	-		Chlorophyll a/b binding protein
Solyc02g070990.1	SL2.50ch02	40517380	40518177	7248.88	2.70	2.55E-007	-	-	-		Chlorophyll a/b binding protein
Solyc02g071000.1	SL2.50ch02	40519780	40520577	7535.60	3.33	3.32E-016	-	-	-		Chlorophyll a/b binding protein
Solyc02g071010.1	SL2.50ch02	40522221	40523018	27284.50	1.34	3.19E-003	-	-	-		Chlorophyll a/b binding protein
Solyc02g071030.1	SL2.50ch02	40525967	40526764	20104.76	0.96	3.02E-002	-	-	-		Chlorophyll a/b binding protein
Solyc02g071080.1	SL2.50ch02	40573395	40574144	39.78	3.21	1.19E-006	-	-	-		serine-type endopeptidase activity
Solyc02g071090.2	SL2.50ch02	40577052	40579474	24.11	3.90	5.47E-006	-	-	-		alpha,alpha-trehalose-phosphate synthase (UDP-forming) activity
Solyc02g071580.2	SL2.50ch02	40975969	40981431	795.76	1.18	1.28E-002	-	-	-		serine-type endopeptidase activity
Solyc02g071700.2	SL2.50ch02	41066412	41069631	75.48	2.49	1.16E-004	-	-	-		hydrolase activity, acting on ester bonds
Solyc02g071730.2	SL2.50ch02	41094016	41099470	11.55	2.21	3.24E-002	-	-	-		AG1, Floral homeotic protein AGAMOUS
Solyc02g071750.2	SL2.50ch02	41119917	41124298	35.79	1.82	1.57E-002	-	-	-		cyclic-nucleotide phosphodiesterase activity
Solyc02g072300.2	SL2.50ch02	41586883	41593725	1135.94	0.80	4.70E-003	-	-	hyper	3-4kb	protein serine/threonine kinase activity
Solyc02g072530.1	SL2.50ch02	41719617	41721053	526.53	1.15	2.91E-002	-	-	-		protein serine/threonine kinase activity
Solyc02g076690.2	SL2.50ch02	41833256	41835924	103.79	1.70	8.85E-004	-	-	hyper	2kb	cysteine-type peptidase activity
Solyc02g077100.2	SL2.50ch02	42161703	42163579	209.03	1.24	4.99E-002	-	-	hyper	4-5kb	triglyceride lipase activity
Solyc02g077110.2	SL2.50ch02	42169831	42171393	212.84	1.37	9.32E-003	-	-	hyper	<1kb	triglyceride lipase activity
Solyc02g077160.2	SL2.50ch02	42187913	42189472	262.44	1.40	1.36E-003	-	-	-		triglyceride lipase activity
